# Standardizing evaluation of patient-specific 3D printed models in surgical planning: development of a cross-disciplinary survey tool for physician and trainee feedback

**DOI:** 10.1186/s12909-022-03581-7

**Published:** 2022-08-12

**Authors:** Lauren Schlegel, Michelle Ho, J. Matthew Fields, Erik Backlund, Robert Pugliese, Kristy M. Shine

**Affiliations:** 1 Jefferson Health Design Lab, 925 Chestnut Street Basement Level, Philadelphia, PA 19107 USA; 2 Sidney Kimmel Medical College of Thomas Jefferson University, 1025 Walnut Street, College Building, Suite 100, Philadelphia, PA 19107 USA; 3 Department of Medicine, Pennsylvania Hospital, University of Pennsylvania Health System, 800 Spruce Street, Philadelphia, PA 19107 USA; 4 Department of Emergency Medicine, Thomas Jefferson University Hospitals, 1020 Sansom Street, Thompson Building, Suite 239, Philadelphia, PA 19107 USA; 5 Innovation Pillar, Thomas Jefferson University Hospitals, 925 Chestnut Street, Suite 110, Philadelphia, PA 19107 USA

**Keywords:** 3D model, 3D printing, Surgical planning, Delphi method, Survey development, Questionnaire

## Abstract

**Background:**

3D printed models are becoming increasingly popular in healthcare as visual and tactile tools to enhance understanding of anatomy and pathology in medical trainee education, provide procedural simulation training, and guide surgical procedures. Patient-specific 3D models are currently being used preoperatively for trainee medical education in planning surgical approaches and intraoperatively to guide decision-making in several specialties. Our study group utilized a modified Delphi process to create a standardized assessment for trainees using patient-specific 3D models as a tool in medical education during pre-surgical planning.

**Methods:**

A literature review was conducted to identify survey questions administered to clinicians in published surgical planning studies regarding the use of patient-specific 3D models. A core study team reviewed these questions, removed duplicates, categorized them, mapped them to overarching themes, and, where applicable, modified individual questions into a form generalizable across surgical specialties. The core study panel included a physician, physician-scientist, social scientist, engineer/medical student, and 3D printing lab manager. A modified Delphi process was then used to solicit feedback on the clarity and relevance of the individual questions from an expert panel consisting of 12 physicians from specialties including anesthesiology, emergency medicine, radiology, urology, otolaryngology, and obstetrics/gynecology. When the Radiological Society of North America (RSNA)/American College of Radiology (ACR) 3D Printing Registry Data Dictionary was released, additional survey questions were reviewed. A final cross-disciplinary survey of the utility of 3D printed models in surgical planning medical education was developed.

**Results:**

The literature review identified 100 questions previously published in surveys assessing patient-specific 3D models for surgical planning. Following the review, generalization, and mapping of survey questions from these studies, a list of 24 questions was generated for review by the expert study team. Five additional questions were identified in the RSNA/ACR 3D Printing Registry Data Dictionary and included for review. A final questionnaire consisting of 20 questions was developed.

**Conclusions:**

As 3D printed models become more common in medical education, the need for standardized assessment is increasingly essential. The standardized questionnaire developed in this study reflects the interests of a variety of stakeholders in patient-specific 3D models across disciplines.

**Supplementary Information:**

The online version contains supplementary material available at 10.1186/s12909-022-03581-7.

## Background

The utilization of three-dimensional (3D) printing has grown dramatically in recent years, bringing new techniques to the healthcare industry. Unlike traditional manufacturing methods (e.g. molding, casting, and subtraction from bulk material blocks), this form of additive manufacturing offers a low-cost, rapid method to generate objects from a digital model or computer-aided design (CAD) file into physical shapes. Moreover, patient-specific data (CT/MRI imaging) can now be processed and printed into 3D form. Applications of 3D printed products in healthcare include tissue and organ fabrication and the creation of customized prosthetics and implants [1]. In addition, 3D printing is being used increasingly to augment medical trainee education, procedural skill acquisition, and treatment planning and decision making in both medicine and surgery. While studies are exponentially increasing, reported measures vary and there exists no simplified, gold standard approach for capturing feedback regarding model features, user experience, and overall utility.

Within graduate medical education and residency training, 3D printing has been used to enhance knowledge and skill acquisition. The ability to rapidly produce detailed mimetic models can be beneficial for visualizing complex anatomy and structural relationships. 3D models have shown benefits to students and trainees in anatomy education and procedure simulation [2, 3]. A meta-analysis done in 2020 of randomized control trials comparing traditional two-dimensional images to 3D printed models found that use of 3D printed models in learning opportunities was associated with higher anatomy exam scores (*p* < 0.05) in post-interventional assessments for medical students [4]. Smerling et al. studied the effect 3D models had on medical students learning complex congenital heart conditions [5]. The students reported increased confidence in understanding complex conditions due to the 3D models and recommended the models for future sessions [5].

The use of patient-specific 3D models for pre-procedure planning has been reported in a variety of surgical and medical specialties. Within cardiology, 3D models have been utilized to visualize the complex, variable pathology of congenital heart diseases and to size implantable devices for left atrial appendage closure, among other uses [6, 7]. Uses in neurosurgery include determining the surgical approach and intraoperative guidance for skull-base tumors and cerebrovascular aneurysms [8–11]. For orthopedic cases, which often involve reconstruction and hardware, 3D models have been used to visualize anatomy, select implant size, and determine drilling trajectories for surgeries treating acetabular defects and scoliosis [12, 13]. Similarly, in otolaryngology and craniomaxillofacial surgery, 3D models have been used to simulate surgery and prebend reconstruction plates for mandibular, orbital, and other craniofacial reconstructions [14–16]. In a 2017 study Marconi et al. reported 3D printed models facilitate a more rapid and clearer understanding of surgical anatomy helping medical students, surgeons, and radiologists spend less time assessing 3D printed models than they would two-dimensional CT scans and 3D virtual reconstructions [17].

In research reporting the uses of 3D models, various measures have been reported to assess the utility of 3D printed models for education and procedure simulation. In a review conducted in 2017 of 93 studies on the use of 3D models for surgical training and simulation, it was found that subjective and objective measures were used in 74 and 61% of studies, respectively [18]. Commonly reported subjective measures include the usefulness of the model, educational value, satisfaction with the model, and confidence [8, 10, 14]. Objective measures reported in studies include procedure time, anesthesia time, operating room time, estimated blood loss, the accuracy of the model, and the correlation between planned and post-operative values [7–9, 12, 13, 15, 19]. Similarly, varied measures have been reported to assess the utility of 3D printed models for surgical planning [20–22]. Yet, there is no consensus on how to measure anatomical accuracy, utility, or user experience with 3D printed models.

We propose the use of a modified Delphi process to review prior measures across specialties to generate a concise, generalizable survey to serve as a gold standard for feedback on the use of 3D printing in surgical planning. The Delphi method is a structured and anonymous process used to collect opinions on a specific topic. It has previously been used in healthcare for a variety of purposes ranging from developing a curriculum for general surgery robotic education to creating a guideline for clinical trial protocol content [23, 24]. The Delphi process consists of the expert evaluation of a questionnaire with variable rounds of review and modification built upon feedback. Here, we modified the Delphi process with categorization/mapping and expert consensus to create a survey containing key questions and language that is broadly applicable across surgical specialties. Though the Radiological Society of North America (RSNA)/American College of Radiology (ACR) recently provided a lengthy, expert consensus-based series of questions to capture multiple elements of 3D printing workflows, no group has created a succinct survey specifically used to assess model fidelity, utility, and trainee experience with 3D models specifically for surgical planning. Moreover, published surveys in this field have not been based on questions developed from systemic analysis of a broad selection of surveys evaluating 3D printed models for surgical planning.

As the use of 3D models for surgical planning expands, there exists a need to more consistently assess the value of such models in trainee development and surgeon experience. Our aim was to provide a standardized survey tool for evaluating the use of 3D models in surgical planning across specialties.

## Methods

### Study team

The core study team consisted of five individuals representing a diversity of professions with experience in the subject matter including a physician, physician-scientist, social scientist, engineer/medical student, and 3D printing lab manager/innovation director. A medical expert panel of 12 individuals was formed representing physicians in anesthesiology, emergency medicine, radiology, urology, otolaryngology, and obstetrics/gynecology. All members of the panel had previous experience working with patient-specific 3D printed models as part of clinical care at the study team’s institution. Participants were recruited through email.

### Literature search

A literature search was conducted in 2019 using the electronic databases Pubmed, Ovid, and SCOPUS. The following keywords were searched in the title and abstract: model OR planning OR training OR education OR teaching OR assessment OR skills OR simulation AND “3D print” OR “3D printing” OR “3D Printed” OR “three-dimensional print” OR “three-dimensional printing” OR “three-dimensional printed”. Articles were screened by reviewing the title and abstract to determine if they met inclusion criteria. Inclusion criteria included 1) published in the English language, 2) primary research (i.e., non-review papers), 3) use of patient-specific 3D printed models for surgical planning, and 4) a questionnaire that assessed surgeon and/or trainee experience. Articles that initially passed inclusion criteria underwent full-text review. Reasons for exclusion after full-text review included: if the literature was a secondary report of the data, non-English, validation study of previously published survey, or solely discussed specific steps of a surgery.

### Data extraction

Articles that met the inclusion criteria were examined and data was extracted from them into a structured tabular form to ensure articles were reviewed consistently. Data extracted included study year, specialty, total survey questions administered, use of objective and subjective questions, type of survey response rating scale used, and each full-text survey question.

### Categorization

To categorize the scope of questions found in the literature extraction, the full-text questions were assigned to one of six categories (anatomy, communication, diagnosis, planning, surgery, or general experience) based on the focus of the question, as determined by the core study team through consensus. Each question was further assigned a construct based on the question’s focus (feasibility, utility, or fidelity). Using the assigned categories and constructs, similar questions were then grouped. Duplicate questions were merged into a single question by consensus of at least two members of the study team. All questions, including both unique and merged questions, were modified into generalized forms that retained the given category and construct but removed references to specific diagnoses or procedures. An example of this categorization process is diagrammed in Table 1.

**Table 1:** Example categorization process

**Study**	**Original Question**	**Category**	**Construct**	**Type**	**Generic Question**
Chen 2018 [25]	Usefulness of 3D prototype for communicating with patients	Communication	Utility	Merged	How much did the 3D model help you with communicating with patients?
Zheng 2018 [19]	How much does the 3D-printing model help you communicate with patients?	Communication	Utility		
Lou 2017 [26]	How critical is the 3D printing model in helping your communication with patients	Communication	Utility		
Aluwee 2017 [27]	Positional relationship between the uterus and the tumor	Anatomy	Fidelity	Merged	How accurate was the region of interest to surrounding structures?
George 2017 [28]	Understand the relationship to vessels	Anatomy	Fidelity		
Wang 2018 [29]	The guides were helpful for precise osteotomy and reduction during surgery	Surgery	Utility	Unique	How helpful was the 3D model for precise surgical maneuvers?

### Mapping

**Fig. 1:**
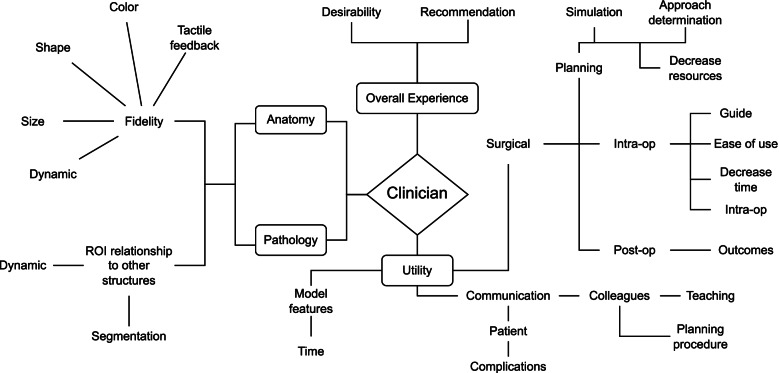
Diagram of mapping process

The core study team reviewed each resultant question from the categorization process. Questions that were too narrow in focus to be applied broadly across surgical specialties and cases as determined by the core study group consensus were removed. Questions too vague to allow consensus in interpretation were also removed. Dominant themes (anatomy, utility, etc.) were determined from the remaining questions, which were then grouped and mapped accordingly until all questions were exhausted (Fig. 1). Each mapped endpoint was then converted into a unique question. The core study team reviewed these questions in the context of known features and clinical use of patient-specific 3D models and considered survey question supplementation if necessary.

### Modified Delphi process

A modified Delphi process was undertaken involving one round of expert feedback and one round of consensus from the core study group to evaluate the question list created by the study team from the literature extraction, categorization, and content mapping process. Questions were evaluated on a 5-point Likert scale for relevance to 3D printing use in presurgical planning and question clarity. Experts were also asked to select whether each question should be retained as-is, reviewed, or removed and to provide free-text comments to explain their recommendations. Individual questions were removed from the question list without further review if they received an average score below 4.0 for relevance (i.e., average expert score was neutral, disagree, or strongly disagree). An example of this process is shown in Table 2.

**Table 2:** Example evolution of a question through the Delphi process

Original survey question	**The model reflects the region of interest’s relationship to other structures** (Strongly disagree = 1, Disagree = 2, Neutral = 3, Agree = 4, Strongly agree = 5)
Feedback questions	- **This question is clear** (Strongly disagree = 1, Disagree = 2, Neutral = 3, Agree = 4, Strongly agree = 5) - **This question is relevant** (Strongly disagree = 1, Disagree = 2, Neutral = 3, Agree = 4, Strongly agree = 5) - **This question should be…** (Retained as-is, Reviewed, Removed) - Comments
Feedback results	- This question is clear – 42% of experts strongly agreed the question was clear (average = 4.2) - This question is relevant – 50% of experts strongly agreed the question was relevant (average = 4.0) - This question should be … - majority of experts (75%) believed the question should be retained as-is (75%)
Feedback comments	- *“perhaps reword: The model reflects anatomic structures’ relationships with each other”* - *“if the model is made without any of those structures, you won’t have any information about surrounding structures”*
Outcome	Original survey question edited to clarify keywords and address comments

Questions with an average score of 4.0 or above for relevance (i.e., experts answered agree or strongly agree) underwent a second round of review and discussion by the core study team. Questions below 4.0 for clarity were reworded by two members of the study team and required consensus from the entire core study team to be included. The review included evaluating relevance and clarity through examination of the expert panel comments. Questions that were determined to be irrelevant or unclear through commentary feedback were removed. Questions where experts noted difficulty interpreting the meaning were reworded by a subgroup of the core study team and sent back to the remainder of the study team for final approval. Once 80% or greater consensus was reached on the new wording of the question, it was finalized.

Concurrently with the development of our survey tool, the 3D Printing Data Dictionary (3DPDD) was released by the RSNA/ACR [30, 31]. This document was developed by expert consensus from members of both professional organizations. The 3DPDD aims to standardize data collection of medical 3D printing outcomes to support efficacy claims and requisite insurance reimbursement for medical 3D printing. This data is being collected by the ACR National Radiology Data Registry (NRDR), 3D Printing Registry. The core study team reviewed all questions in the data registry and selected only those relevant to clinician use of patient-specific 3D models. These questions were then compared against the question list generated after the mapping process. Duplicates and/or questions not generalizable across specialties were removed. The remaining questions from the RSNA/ACR list were modified for brevity and added to our survey.

After the final survey was developed, readability was measured using both the Flesch-Kincaid Grade Level and FORCAST grade level [32–34]. The length of the survey was calculated to ensure appropriate brevity [35]. After preliminary data was collected, Cronbach’s alpha was used to measure reliability in each category of the survey (anatomy, utility, and experience).

## Results

### Literature search

The literature review of Pubmed, Ovid, and SCOPUS identified 7141 total studies of which 3705 studies remained after duplicates were removed. After screening titles and abstracts for face validity, 116 studies were selected for full-text review to determine which met the inclusion criteria. Following a full-text review, 100 articles were excluded including 18 that were focused on surgical simulation and 65 that did not administer a questionnaire or survey. Additional causes for exclusion were if the paper was not the primary report of the data, if it was not written in English, if it was related to the validation of a surgical tool, or if it was related to learning the specific steps of a surgery. As a result, 16 articles were included in the final dataset for question extraction. The studies used in the survey were published between 2012 and 2019. Within the 16 articles, five surgical specialties were represented. The most represented surgical specialty was orthopedics (*n* = 9), whereas other specialties were represented singly or at maximum in duplicate (Table 3).

**Table 3:** Represented specialties in included journal articles

Specialty	Number of Articles
Orthopedics	9
General Surgery	2
Urology	2
Cardiology	1
Obstetrics-Gynecology	1
Otolaryngology	1

Of the research studies included in the dataset, none noted the completion of a validation process in the study methods to develop the survey questionnaire administered. Two studies utilized surveys that were previously published. The median number of questions per individual survey among the dataset was 5 (range 1–11). A total of 100 full-text questions were extracted from the articles. Half of the studies (*n* = 8) reported on both subjective and objective measures while the other half (*n* = 8) reported on subjective measures only. There were no studies that reported on objective measures only. Rating scales for survey responses varied among the 16 articles and included 10 point (*n* = 8), 6 point (*n* = 1), 5 point (*n* = 2), 5 point Likert (*n* = 2), and 4 point scales (*n* = 1).

Nine studies (56%) administered questions evaluating the anatomic accuracy of the 3D models. Five studies (31%) assessed whether the 3D model helped the surgeon communicate in general with patients, whereas two studies (12.5%) asked specifically whether the 3D models helped explain the procedure to patients. Of the included studies, nine (56%) asked users to rate whether the model was helpful for preoperative planning.

The most commonly asked questions were whether the 3D model accurately reflected the region of interest (ROI) anatomy (56%) and whether the model was helpful in preoperative planning (56%). While each of these questions was asked by over half of the included studies, only six (37.5%) studies asked both questions.

### Categorization

Of the 100 full-text survey questions extracted, 33 questions were categorized as pertaining to anatomy, 24 relating to surgery, 18 concerning planning, 12 assessing general experience, 11 exploring communication, and two representing diagnosis. Following the sorting and categorization of each question, 55 questions were noted to substantively duplicate at least one other question within its category. Duplicate questions were reviewed, merged, and re-written in generic form yielding 45 unique representative questions in the dataset (Table 4).

**Table 4:** Questions in the dataset by category pre and post duplicate question merging

Category Assigned	Number of Questions	Number of Questions (merging duplicates)
Anatomy	33	15
Communication	11	5
Diagnosis	2	1
Experience	12	4
Planning	18	7
Surgery	24	13

### Mapping

After a review of unique questions and discussion by the study team, tactile feedback was the only model feature not captured in the dataset, prompting the addition of a question addressing it, using language derived from previous studies for model features. A set of 24 questions was thus generated (Appendix). These questions were re-categorized (anatomy, utility, and experience) to reflect the themes present across the questions.

### Modified Delphi process

For the modified Delphi process, 15 clinicians with previous experience in 3D printed models for clinical care were recruited through email. Twelve experts (80% response rate) participated in a review of the 24 questions generated during the previous mapping session. Of the 10 initial anatomy questions, three questions received an average score below 4.0 for relevancy and were removed. Seven questions scored 4.0 or above for relevancy and underwent further review. Of these seven questions, four questions were mentioned to address similar concepts in feedback and were combined into two questions. The core study team added one question from the 3DPDD and another to reflect feedback from the Delphi process, yielding a total of seven anatomy questions in the final survey.

A total of 12 questions related to the utility of patient-specific 3D models were reviewed by the experts and none scored below 4.0 for relevancy. After review, five questions were removed from the utility question pool, four for redundancy and one was moved for consideration in the experience section. One question was noted to be similar to a question in the 3DPDD and a modified version of the latter was used. After review of the expert feedback by the core study team, there were a total of seven utility questions.

Three questions relating to the experience of using patient-specific 3D models were reviewed by the experts as all scored above 4.0 for relevancy. All three of these were kept. Additionally, there were three questions from the 3DPDD that were added to this section in a modified succinct format agreed upon by consensus of the core study team. The final survey is listed in Table 5 and each question should be evaluated on a Likert scale.

**Table 5:** Final survey questions

Anatomy
1. The quality of the model was adequate* 2. The resolution of the model was adequate 3. The model coloring helped identify relevant structures 4. The model accurately reflected the patient’s anatomy in size 5. The model accurately reflected the patient’s anatomy in shape 6. The model features reflected the region of interest’s relationship to other relevant structures 7. The model helped identify pathology
Utility
1. The model was useful for trainee education 2. The model was useful for procedure planning 3. The model was useful during the procedure 4. The model was useful for communication with patients 5. Use of the model saved me time* 6. Use of the model decreased overall supplies related to the procedure 7. Use of the model revised the patient’s treatment plan*
Experience
1. The model was easy for me to use* 2. The model met my needs 3. The model was important in this case* 4. I would use a patient-specific 3D model in the future 5. I would recommend use of a patient-specific 3D model in the future 6. After using the 3D printed model, I was more confident in the treatment plan*

^*﻿^questions derived from 3DPDD

#### Initial evaluation of final survey

After the final survey was developed, readability was measured. The Flesch-Kincaid Grade Level was found to be 8.3 and the FORCAST grade was 12.3. The length of the survey was calculated to be around 4 min. Preliminary data suggests a response rate of 74% across 38 cases in which 107 surveys were sent out. Cronbach’s alpha was calculated for each category within the survey and found to show good internal consistency (anatomy, α = 0.725; utility α = 0.736; experience α = 0.779). However, more data is necessary to fully validate the survey.

## Discussion

We have described a multi-disciplinary approach to creating a survey that quantifies the experience of using a patient-specific 3D model for medical education in surgical planning. In the literature review performed by the study team, the majority of studies selected for full-text review were excluded for not administering a questionnaire [36–38]. This suggests the need for a standardized assessment tool to help produce robust 3D printing research. Of the included studies, the most common types of unique questions were related to anatomy and surgical planning, while questions relating to communication and experience using 3D models were less common. The results of our Delphi process also reflected the preference towards surveying the anatomy and utility of 3D printing models. However, there is significant heterogeneity between studies when assessing 3D model performance.

Our approach builds on previous literature, employing both quantitative and qualitative methodology in the Delphi process. By utilizing the results of an extensive literature review, this method provides data already evaluated by those with experience in 3D printing. The use of an anonymous feedback technique to develop a survey to assess 3D printing has not been reported previously. The Delphi method has been used and validated in several fields, including healthcare [24, 39–41]. Given the role of user feedback, the output of the Delphi method is dependent on the selection of expert participants. Our team of 3D printing experts consisted of clinicians with previous experience utilizing patient-specific 3D models for clinical care. Feedback from this expert group is likely biased towards experienced users and therefore does not incorporate the thoughts of inexperienced users. Potential users who are new to 3D printing may have different questions and needs for patient-specific 3D models. Additionally, our study was limited to a core study team and expert panel practicing at a single institution utilizing the same core 3D printing technologies. Users with different equipment and resources may have distinct needs.

Compared to other survey development methods, the Delphi process utilizes the feedback from an expert panel. An expert is generally considered someone with experience and knowledge of the field who is well-respected [40]. Our study was conducted at an academic hospital and consisted of physicians and administrators with experience in medical 3D printing. As a result, the results reflect aspects of 3D printing and models that may not be as relevant to community hospitals, such as teaching and simulation. To allow for these differences, the option of “not applicable” was included as a possible response. While we recognize not all facilities utilizing 3D models have trainees, these questions were reported frequently in the literature. Thus, we felt questions related to trainee education were relevant to include, with the option to not answer.

To create a more manageable survey for the modified Delphi process, the study team reviewed, categorized, and mapped the generalized survey questions found through the literature search that were used in previously published research of 3D printed models. Through the mapping process the number of potential questions decreased from 45 to 24 and created shorter, clearer questions. In keeping with the goal of the study to develop a generalizable survey tool to standardize 3D printing model feedback, questions found to be too specific, long, or vague were removed or reworded. Another reason this was done was to eliminate survey fatigue - a common problem in survey data collection. Survey length, along with question complexity and question type, are known to influence survey response [42]. Given these factors, our goal was to create a survey that takes less than 5 min. The study team worked to limit the number of questions in the final questionnaire, arriving at an estimated survey length of about 4 min. We aimed to develop a survey that was easily understandable even if the respondents are highly educated and readability will not likely be a deterrent. Scoring a Flesch-Kincaid Grade Level of 8.3 speaks to the ease of reading and while the FORCAST grade was 12.3, which is above the recommendation for the general public, we accepted this score as all of our readers are physicians who complete at least 8 years post-high school education. Additionally, we chose the Likert scale as a way to create semi-quantitative data that could be compared across multiple studies.

The release of the 3DPDD during the study period, after the Delphi feedback from our expert panel, influenced the final survey development. The Data Dictionary is composed of ten sections (Patient, Order, Digital Modeling, Printer, 3D Printed Model, Procedure, Effort, User Assessments, Outcome Assessments, and Outcomes) with a total of 112 questions. The User Assessments section is directed towards clinical users of 3D models and consists of eight questions with Likert scale responses. In reviewing these questions, the core study team found that five were different from questions developed through the Delphi process, of which three related to the experience of using patient-specific 3D models. While these five questions did not undergo separate Delphi review by the expert panel, the core study team determined these questions were important to add by consensus, given that the 3DPDD and 3D Printing Registry were developed through expert agreement and leadership within these organizations. By including these questions in our survey, we hope it will promote the contribution of data into the 3D Printing Registry. Additionally, including the RSNA/ACR data in the final survey has improved the balance of emphasis between anatomy, utility, and experience.

The 3DPDD is both comprehensive and generalizable, however, it is not designed as an off-the-shelf survey tool and does not include some questions that we found valuable for collecting information for the understanding of subjective claims of satisfaction and efficacy relating to both surgical planning and patient communication. Therefore, we found it important to continue generating this survey tool to be a readily deployable asset that augments the efforts of the RSNA/ACR and fills a need gap.

In the initial review of the survey, a response rate of 74% was achieved across the 107 surveys sent out to a population of academic physicians indicating a positive majority response. Additionally, good reliability was noted across all categories of the survey with each category scoring above 0.700 for Cronbach’s alpha. Our future work will continue the collection of data to further validate the survey and report on data captured by the survey questions. 

## Conclusion

This study illustrates how a literature review-based Delphi method can be used to produce a generalized survey to quantify the experience of using a patient-specific 3D model for surgical planning and trainee education. Through this anonymous feedback process, the study team engaged a variety of stakeholders across an institution to create a survey that can be used to track the use of 3D models. The strength of this survey tool is in that it allows for the comparison of 3D model features and utility in pre-surgical planning across institutions, practices, and specialties.

The study team has generated a survey informed by quantitative and qualitative data collection that reflects the diverse needs and experiences of clinicians across healthcare specialties when utilizing patient-specific 3D models. As a valuable educational tool, 3D printed models are utilized and studied for surgical planning at an increasing rate. In the future, data from surveys will contribute evidence to the medical community in a standardized fashion to inform the design, utility, and experience of patient-specific 3D models in surgical planning.

## Supplementary Information

**Additional file 1.**

## Data Availability

The datasets used and/or analyzed during the current study are available from the corresponding author on reasonable request.

## Abbreviations

RSNA: Radiological Society of North America
ACR: American College of Radiology
3DPDD: 3D Printing Data Dictionary
NRDR: National Radiology Data Registry
