# Supplementary material for: Standardizing evaluation of patient-specific 3D printed models in surgical planning: development of a cross-disciplinary survey tool for physician and trainee feedback
Source: BMC Med Educ. 2022 Aug 12;22:614. doi: 10.1186/s12909-022-03581-7 (PMC9373487; doi:10.1186/s12909-022-03581-7)
Supplement: Supplementary file 1 — Additional file 1. [file 12909_2022_3581_MOESM1_ESM.docx]

Appendix

Questions from RSNA 3D SIG Data Registry not included

805 Before using the 3D printed model, I was confident in the treatment plan

810 Use of the 3D printed model or guide was compatible with other aspects of my approach to this case

Questions posed to expert panel prior to modification in Delphi process

| Anatomy |
| --- |
| 1. The model's colors help identify structures 2. The model's shape is accurate 3. The model's size is accurate 4. The model's tactile feedback is accurate 5. The model's interactive features are functionally relevant 6. The model reflects the region of interest's relationship to other structures 7. The model's interactive features reflect the region of interest's relationship to other structures 8. The region of interest's colors help identify the pathology 9. The region of interest's shape is reflective of the pathology 10. The region of interest's size is reflective of the pathology |
| Utility |
| 1. The model was useful for simulation teaching 2. The model was useful for procedure planning 3. The model was useful for communicating about the procedure with patients 4. The model was produced in adequate time 5. I am satisfied with the model 6. The model was useful during the procedure as a guide 7. The model was easy to use during the procedure 8. The model helped to decreased procedure time 9. The model helped to decrease supplies used during the procedure 10. The model was useful for pre-procedure simulation 11. The model was useful to determine approach 12. The model helped to decrease pre-procedure resources used |
| Experience |
| 1. I would use a patient-specific 3D model in the future for this procedure 2. I recommend use of a patient-specific 3D model in the future for this procedure |
